# Cortical microstructure in primary progressive aphasia: a multicenter study

**DOI:** 10.1186/s13195-022-00974-0

**Published:** 2022-02-09

**Authors:** Ignacio Illán-Gala, Victor Montal, Sergi Borrego-Écija, Maria Luisa Mandelli, Neus Falgàs, Ariane E. Welch, Jordi Pegueroles, Miguel Santos-Santos, Alexandre Bejanin, Daniel Alcolea, Oriol Dols-Icardo, Olivia Belbin, Mª. Belén Sánchez-Saudinós, Nuria Bargalló, Sofía González-Ortiz, Albert Lladó, Rafael Blesa, Bradford C. Dickerson, Howard J. Rosen, Bruce L. Miller, Alberto Lleó, Maria Luisa Gorno-Tempini, Raquel Sánchez-Valle, Juan Fortea

**Affiliations:** 1 Memory Unit, Department of Neurology, Hospital de la Santa Creu i Sant Pau, Biomedical Research Institute Sant Pau, Sant Antoni Maria Claret, 167, 08025 Barcelona, Spain; 2 Centro de Investigación Biomédica en Red de Enfermedades Neurodegenerativas (CIBERNED), Barcelona, Spain; 3 Atlantic Fellow for Equity in Brain Health at the University of California San Francisco, San Francisco, CA 94115 USA; 4 Alzheimer’s Disease and Other Cognitive Disorders Unit, Service of Neurology, Hospital Clínic de Barcelona, Institut d’Investigació Biomèdica August Pi i Sunyer, University of Barcelona, 08036 Barcelona, Spain; 5 Memory and Aging Center, Department of Neurology, University of California, San Francisco, CA 94115 USA; 6 Radiology Department, Hospital Clinic Barcelona and Magnetic Resonance Image Core facility, Institut d’Investigacions Biomèdiques August Pi i Sunyer (IDIBAPS), Barcelona, Spain; 7 Department of Radiology, Hospital del Mar, Barcelona, Spain; 8 Department of Neurology, Massachusetts General Hospital and Harvard Medical School, Boston, MA USA; 9 Massachusetts Alzheimer’s Disease Research Center, Boston, MA USA; 10 Barcelona Down Medical Center. Fundació Catalana de Síndrome de Down, Barcelona, Spain

**Keywords:** Diffusion, Magnetic resonance, Primary progressive aphasia, Alzheimer’s disease, Frontotemporal lobar degeneration

## Abstract

**Background:**

Cortical mean diffusivity is a novel imaging metric sensitive to early changes in neurodegenerative syndromes. Higher cortical mean diffusivity values reflect microstructural disorganization and have been proposed as a sensitive biomarker that might antedate macroscopic cortical changes. We aimed to test the hypothesis that cortical mean diffusivity is more sensitive than cortical thickness to detect cortical changes in primary progressive aphasia (PPA).

**Methods:**

In this multicenter, case-control study, we recruited 120 patients with PPA (52 non-fluent, 31 semantic, and 32 logopenic variants; and 5 *GRN*-related PPA) as well as 89 controls from three centers. The 3-Tesla MRI protocol included structural and diffusion-weighted sequences. Disease severity was assessed with the Clinical Dementia Rating scale. Cortical thickness and cortical mean diffusivity were computed using a surface-based approach.

**Results:**

The comparison between each PPA variant and controls revealed cortical mean diffusivity increases and cortical thinning in overlapping regions, reflecting the canonical loci of neurodegeneration of each variant. Importantly, cortical mean diffusivity increases also expanded to other PPA-related areas and correlated with disease severity in all PPA groups. Cortical mean diffusivity was also increased in patients with very mild PPA when only minimal cortical thinning was observed and showed a good correlation with measures of disease severity.

**Conclusions:**

Cortical mean diffusivity shows promise as a sensitive biomarker for the study of the neurodegeneration-related microstructural changes in PPA.

**Supplementary Information:**

The online version contains supplementary material available at 10.1186/s13195-022-00974-0.

## Introduction 

Primary progressive aphasia (PPA) encompasses different neurodegenerative syndromes characterized by prominent deterioration of speech and language, and relative sparing of other cognitive functions [1, 2]. In 2011, an international group of experts introduced a common framework in which three variants of PPA were recognized, based on specific speech and language features: the non-fluent agrammatic variant (nfvPPA), the semantic variant (svPPA), and the logopenic (lvPPA) variant [3].

Currently, there are no disease-modifying treatments for PPA, but many pharmacological and non-pharmacological treatments are being developed with promising results [4]. However, several factors hamper the design of clinical trials in PPA. First, PPA cases are relatively rare, limiting the recruitment of participants [5]. Second, the diagnosis of PPA is difficult at very mild stages, when disease-modifying interventions are more likely to be effective, but atrophy can be subtle [6]. Finally, the prognosis of each PPA variant is heterogeneous and the progression rate can vary substantially within a given PPA variant [7, 8]. In this complex scenario, biomarkers represent powerful tools for the design of clinical trials [7]. Particularly, future clinical trials would largely benefit from instruments sensitive to the earliest neurodegenerative changes to increase diagnostic certainty, and optimize the measurement of treatment effects [7, 8].

Quantitative analysis of brain structure with magnetic resonance imaging (MRI) can detect disease-specific abnormalities and enable the comparison with normal populations and the determination of the vulnerable regions in each syndrome. Previous MRI studies have shown that each PPA variant is characterized by a relatively focal onset, and spreading along highly connected cerebral regions [9–12]. To date, most neuroimaging studies in PPA have focused on the cortical macrostructure with different metrics (gray matter density in voxel-based morphometry studies or cortical thickness in surface-based analyses) or white matter microstructural properties (namely diffusion tensor imaging metrics such as fractional anisotropy). However, diffusion tensor imaging can also be used to measure the magnitude of diffusivity (mean diffusivity), in the cerebral cortex [13]. Higher cortical mean diffusivity values reflect microstructural disorganization and disruption of cellular membranes and have been proposed as a sensitive biomarker that might antedate macroscopic cortical changes [13, 14]. We have previously shown that diffusion imaging can also be used to measure the structural organization of the cerebral cortex and that such changes may reveal neurodegeneration that is not detected with cortical thickness measurements in different neurodegenerative diseases [14–16], even in the preclinical phase [17, 18].

In this multicenter study, our objective was to compare the cortical thickness and cortical mean diffusivity changes in patients with PPA. We also aimed to correlate these changes with clinical measures of disease severity. We hypothesized that cortical mean diffusivity is more sensitive than cortical thickness to detect cortical changes in PPA.

## Materials and methods

### Study population

Participants with PPA were recruited from two cohorts, at three different centers: 88 at the Memory and Aging Center (MAC) of University of California, San Francisco (UCSF, CA, USA), and 72 at the Catalan Frontotemporal Dementia Initiative (CATFI; 32 at Hospital de Sant Pau and 40 at the Hospital Clinic de Barcelona, Barcelona, Spain). In both cohorts, the diagnosis was made by neurologists with expertise in the evaluation of PPA [19, 20]. All patients underwent a complete clinical history, physical examination, neuropsychological evaluation, genetic screening for major FTLD mutations (for additional details, please refer to the “Genetic studies, biomarkers, and pathological assessment” section) and structural brain imaging [15, 21, 22]. A total of 92 age-matched healthy controls from the two cohorts were also included as imaging controls (35 UCSF and 57 CATFI). All healthy controls had normal cognitive performance according to normative data and did not have any neurologic, psychiatric, or other major medical illnesses [20, 23].

**Fig. 1:**
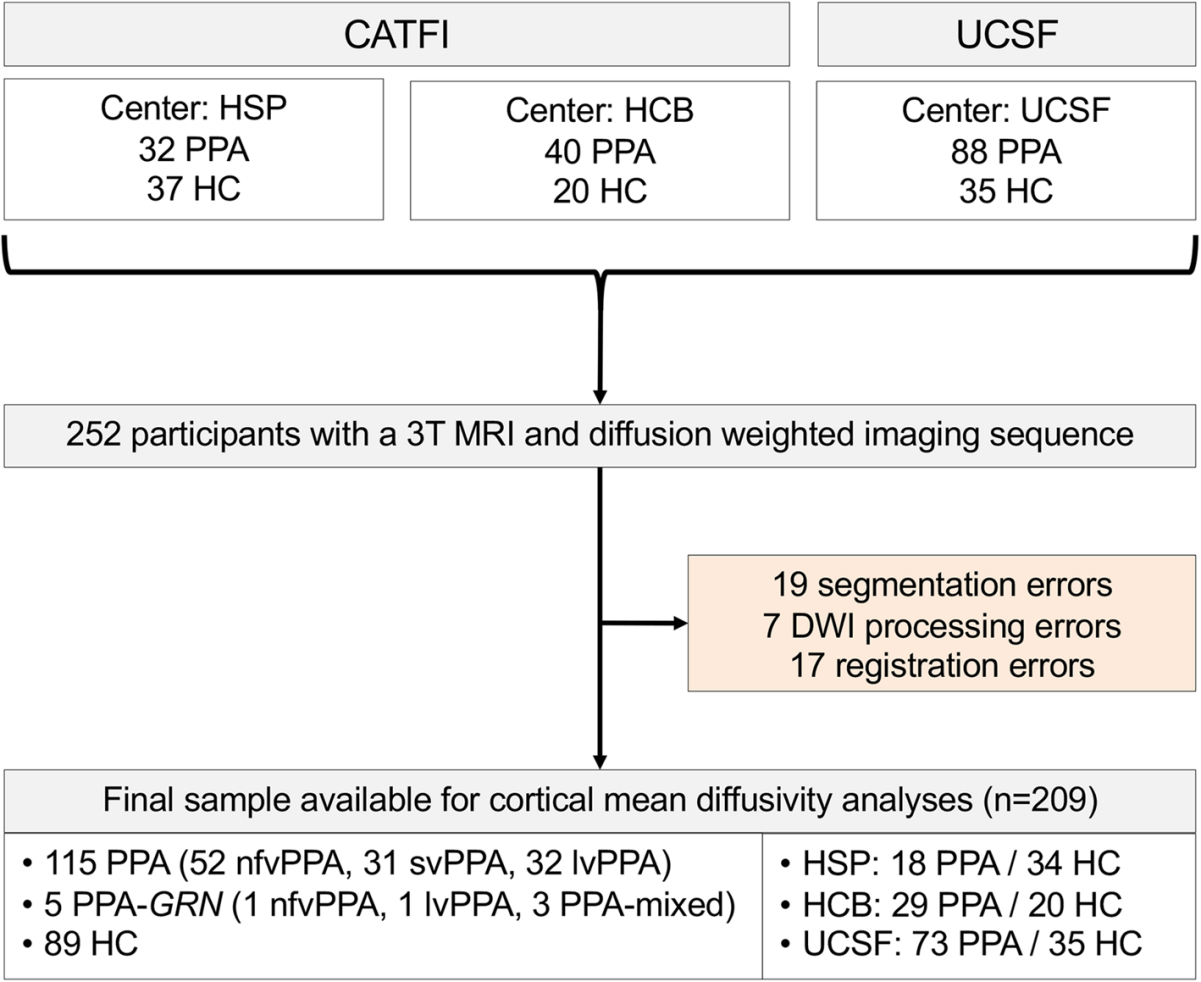
Flowchart of the sample composition. Abbreviations: CATFI, Catalan Frontotemporal Dementia Initiative; FTLD, frontotemporal lobar degeneration; HC, healthy controls; lvPPA, logopenic variant of primary progressive aphasia; MRI, magnetic resonance image; nfvPPA, non-fluent/agrammatic variant of primary progressive aphasia; PPA, primary progressive aphasia; svPPA, semantic variant of primary progressive aphasia; UCSF, University of California San Francisco

Inclusion criteria for PPA participants were (i) meeting basic PPA criteria as defined by Mesulam [1], (ii) meeting international criteria for one of the three PPA variants [3], and (iii) 3T MRI study available for structural and cortical mean diffusivity analysis (see below for details). Participants fulfilling basic PPA criteria that were found to have a mutation in the *GRN* gene (*n* = 5) were classified in a separate group because these cases are characterized by a widespread and asymmetric pattern of gray matter loss [24, 25] and usually display clinical features of more than one PPA subtype [26]. Figure 1 shows a flowchart of the sample composition. A total of 252 participants with appropriate 3T structural and diffusion-weighted MRI were considered for analysis. Of these, 43 (17%) participants were excluded due to quality control issues or processing errors.

### Measures of disease severity and global cognitive function

At presentation, the CDR Dementia Staging Instrument plus National Alzheimer’s Coordinating Center Behavior and Language Domains sum of boxes (CDR® plus NACC FTLD-SB) scores were recorded as a measure of global of disease severity [27, 28]. The CDR® plus NACC FTLD-SB was designed to apply the CDR® in FTLD natural history studies. It includes cognitive/functional domains: memory, orientation, judgment and problem solving, community affairs, home and hobbies, personal care, behavior, and language. Each category domain is rated on a 5-point scale ranging from 0 (normal), 0.5 (questionably or minimally impaired), 1 (mildly but definitely impaired), 2 (moderately impaired), to 3 (severely impaired). All ratings are based on the semi-structured interview from both a patient and a knowledgeable informant (typically a close family member of a patient) and do not consider neuropsychological assessments. The CDR® plus NACC FTLD-SB score (ranging from 0 to 24) is obtained by adding the rating of each domain. The global CDR® plus NACC FTLD (ranging from 0 to 3) score was also calculated following recently published scoring rules [28] to identify PPA participants at a mild stage of disease (global CDR® plus NACC FTLD score of 0.5). CDR® plus NACC FTLD-SB was available in 183 (88%) of participants. The Mini-Mental State Examination (MMSE) was recorded in all centers as a general measure of cognitive impairment [29].

### Genetic studies, biomarkers, and pathological assessment

*APOE* was genotyped in all participants according to previously described methods [30]. Patients were screened for genetic mutations known to cause frontotemporal lobar degeneration and Alzheimer’s disease (AD) [15, 21]. Genetic screening was conducted for mutations known to cause autosomal dominant FTLD or AD (*MAPT*, *C9orf72*, *GRN*, *TARDBP*, *FUS*, *PSEN1*, *PSEN2*, and *APP*) at each site, as previously described [15, 21].

Amyloid PET and core AD biomarkers were also available in a subset of participants. Amyloid PET studies were read as positive or negative, as previously described [20], and were performed with two different tracers ^11^C-Pittsburgh compound B at UCSF [*n* = 37] and with ^18^F-Florbetapir at CATFI [*n* =4]). The core AD biomarkers were available for CATFI participants and were performed as previously described [20, 31].

Neuropathologic assessments performed at UCSF or Barcelona’s Neurological Tissue Bank followed previously described procedures [20, 21]. Participants were classified into FTLD major molecular classes (tau, TDP-43, or FUS) and subtypes [22] or AD [32].

### MRI

#### MRI acquisition

All participants underwent a 3T MRI using 4 different scanners. The acquisition parameters by the scanner can be found in Supplementary Table 1-2. All centers had a structural 3D MPRAGE T1-weighted acquisition of approximately 1 × 1 × 1 mm isotropic resolution and an EPI diffusion-weighted acquisition of at least 2.7 × 2.7 × 2.7 mm isotropic resolution.

#### Cortical thickness processing

Cortical thickness reconstruction was performed with the Freesurfer package v6 (“FreeSurfer,” n.d.) using a previously described procedure [33]. All individual cortical reconstructions were visually inspected on a slice-by-slice basis to check for accuracy of the gray/white matter boundary segmentation. From the initial 252 participants with 3T MRI available from the three centers, 19 were excluded due to segmentation issues. Finally, each individual reconstructed brain was registered, and cortical thickness maps were morphed, to the fsaverage standard surface provided by Freesurfer, using a spherical registration, enabling an accurate inter-subject matching of cortical locations for the computation of further statistics. Before statistical analyses, we smoothed the cortical thickness maps using a Gaussian kernel with FWHM of 15mm as implemented in Freesurfer [34].

#### Cortical mean diffusivity processing

We used a previously described home-made surface-based approach to process cortical diffusion MRI [14]. Recent studies have shown the potential of surface-based methods to measure microstructural changes in neurodegenerative diseases [35] and the cortical architecture [36]. An important advantage of these methods is the mitigation of partial volume effects or kernel-sensitive CSF signal inclusion during the smoothing step [37]. Briefly, diffusion-weighted imaging data were first corrected for motion effects applying a rigid body transformation between the *b* = 0 image and the diffusion-weighted acquisitions. Then, after removing non-brain tissue using the Brain Extraction Tool, diffusion tensors were fitted, and mean diffusivity was computed using the FSL’s dtifit command. We then computed the affine transformation between the skull-stripped b0 and the segmented T1-weighted volume using a boundary-based algorithm as implemented in Freesurfer’s bbregister. This approach takes advantage of the accurate segmentation of the white matter surface and pial surface obtained during Freesurfer’s segmentation (cortical thickness processing section), to accurately register the b0 and the T1-weighted image, maximizing the intensity gradient across gray matter and white matter between both volumes. At this point, all the diffusion to T1 registrations were visually inspected to exclude those subjects with an erroneous co-registration. Seven participants were excluded due to diffusion-weighted imaging processing errors. Then, the mean diffusivity volume for each individual was sampled at the midpoint of the cortical ribbon (half the distance along the normal vector between the white matter surface and the gray matter surface) and projected to each surface reconstruction obtained during the Freesurfer processing, to create a surface map of cortical mean diffusivity (using Freesurfer’s mri_vol2surf command). Finally, individual cortical mean diffusivity maps were normalized to an average standard surface using a spherical registration, enabling an accurate inter-subject matching of cortical locations for the statistical analyses. Before statistical analyses, we applied a Gaussian kernel of 15 mm as implemented in Freesurfer [34], to obtain equivalent data effective smoothing between cortical thickness and cortical mean diffusivity.

#### Cortical mean diffusivity harmonization between centers

Because diffusion tensor imaging metrics are very sensitive to acquisition parameters, harmonization approaches are required to mitigate center-specific differences in multicenter studies. We applied a multicenter harmonization algorithm based on ComBat, to reduce center-specific differences in cortical mean diffusivity quantifications before any statistical analysis [38]. Briefly, ComBat uses an empirical Bayes framework to estimate the additive (mean) and multiplicative (variance) contribution of each site, at each vertex, for a specific diffusion tensor imaging metric and corrects these effects. Importantly, this approach allows the inclusion of biological information (such as clinical group or age), and it has been reported to preserve within-site biological variability, thereby increasing the statistical power [38].

### Statistical methods

Data were explored for normality using the Shapiro-Wilk test. When necessary, variables were log-transformed using the natural log to fulfill the normal distribution assumption. Between-group differences were determined with ANOVA or *t*-test for continuous variables (with Bonferroni correction for multiple comparisons) and the chi-square for dichotomous or categorical data. We first performed group comparisons for cortical thickness and cortical mean diffusivity with a two-class general linear model, as implemented in Freesurfer, comparing each PPA variant to the control group. These analyses were repeated for each center independently. Moreover, we also performed additional analyses including only PPA participants with a global CDR® plus NACC FTLD score of 0.5 (mild PPA). Next, we performed a vertex-wise correlation analysis in each PPA variant group between the cortical thickness and cortical mean diffusivity and the CDR® plus NACC FTLD-SB (as a general measure of disease severity) and the MMSE (as a general measure of cognitive function). Specifically, a general linear model was created in which cortical mean diffusivity or cortical thickness was included as the dependent variable, and CDR® plus NACC FTLD-SB scores (and MMSE scores) were independent variables. We included age, sex, handedness, and center as nuisance variables in the cortical thickness analysis. In mean diffusivity analysis, only age, sex, and handedness were included since diffusion tensor imaging data were already harmonized between centers in a previous step. Only results that survived multiple comparisons (family-wise error < 0.05) based on Monte Carlo simulation with 10,000 repeats as implemented in Freesurfer are presented. We used a very stringent threshold of *α* = 0.001 for the group analyses including all PPA participants and HC, and a threshold of *α* = 0.05 for the analyses including PPA subgroups and the correlation analyses. A full description of the multiple comparison’s methodology can be found in the Supplementary material.

We computed the Cohen’s *d* effect size metric for both cortical thickness and cortical mean diffusivity, on a vertex-wise basis, to obtain a topographical representation of the effect size for the group comparison between participants with PPA and controls. Effect size computation was restricted to cortical regions showing statistically significant differences between participants with PPA and controls for either cortical thickness or cortical mean diffusivity. We then computed the difference between the cortical thickness and cortical mean diffusivity effect size maps to obtain a topographical representation of the net effect size for each metric.

## Results

Table 1 shows the demographics and clinical features of the participants in the study. Age at MRI, years of education, and disease duration at MRI were similar between participants with PPA and controls and between PPA subgroups. Sex and handedness distribution were also similar between all groups. As expected, all PPA variants showed lower values of MMSE and CDR® plus NACC FTLD-SB scores compared to the control group. Participants in the lvPPA group had lower MMSE scores when compared to participants in the nfvPPA and PPA-*GRN* groups. APOEɛ4 carriers were overrepresented in the lvPPA group (33%) compared to the nfvPPA, svPPA, and GRN-PPA groups (19%, 16%, and 0%, respectively). The CDR® plus NACC FTLD-SB scores were higher in the svPPA group when compared to the nfvPPA group. The frequency of PPA participants with a global CDR® plus NACC FTLD score of 0.5 ranged from 19% in svPPA to 38% in nfvPPA.

**Table 1:** Characteristics of the participants

	PPA					HC
	nfvPPA ***n***=52 (43%)	svPPA ***n***=31 (26%)	lvPPA ***n***=32 (27%)	PPA-GRN ***n***=5 (4%)	All PPA ***n***=120 (100%)	HC ***n***=89
**Cohort, no. (CATFI/UCSF)**	18/34	15/16	11/21	3/2	47/73†	54/35†
**Demographics, genetic, and clinical features**						
Age at MRI, years	69.0 (7.3)	67.1 (7.5)	66.3 (7.9)	61.7 (3.3)	67.5 (7.5)	66.0 (6.9)
Biological sex (men/women)	20/32	16/15	16/16	0/5	52/68	31/58
APOEɛ4, no. (positive/negative)	9/39	4/21	9/18	0/4	22/82	7/28
Education, years	14.2 (5.2)	14.6 (4.0)	13.1 (7.2)	12.4 (5.7)	13.9 (5.5)	14.7 (4.4)
Handedness (right/left)	45/7	28/3	29/3	4/1	106/14	85/4
Disease duration at MRI, years	4.2 (2.3)	4.5 (3.2)	4.1 (1.8)	2.5 (1.6)	4.2 (2.4)	-
**Measures of cognition and disease severity**						
MMSE, /30	25.0 (4.6)‡	23.9 (4.6)	20.7 (6.2)‡	27.0 (2.6)‡	23.7 (5.3)†	29.1 (0.9)†
CDR® plus NACC FTLD-SB, /24^a^	3.9 (2.6)§	6.3 (3.2)§	5.1 (2.8)	3.8 (4.6)	4.9 (3.0)†	0.0 (0)†
Global CDR® plus NACC FTLD = 0.5, no. (%)	20 (38)	6 (19)	9 (28)	1 (20)	36 (30)†	0 (0)†
**Biomarkers and autopsy findings**						
Amyloid PET, no. (positive/negative)	0/21	1/6	13/0	0/0	14/27	1/1
AD biomarkers in CSF, no. (Positive/Negative)^b^	3/12	2/12	9/0	0/2	14/26	5/27
Pathological diagnosis, no. (FTLD/AD)^c^	16/0	3/0	0/8	2/0	21/8	0/0

*Abbreviations*: *AD*, Alzheimer’s disease; *CATFI*, Catalan Frontotemporal Dementia Initiative; *CDR*, Clinical Dementia Rating; *CDR® plus NACC FTLD-SB*, CDR Dementia Staging Instrument plus National Alzheimer’s Coordinating Center Behavior and Language Domains sum of boxes; *MRI*, magnetic resonance image; *MMSE*, Mini-Mental State Examination; *UCSF*, University of California San Francisco; *lvPPA*, logopenic variant of primary progressive aphasia; *nfvPPA*, non-fluent/agrammatic variant of primary progressive aphasia; *svPPA*, semantic variant of primary progressive aphasia

^a^CDR® plus NACC FTLD-SB was available in 183 (88%) of participants (94 PPA and 89 HC)

^b^A positive Alzheimer’s disease biomarker profile was defined by an abnormal Aß1-42 to Aß1-40 ratio and a total-tau to Aß1-42 ratio

^c^The 21 FTLD cases included 6 cases with corticobasal degeneration (all in the nfvPPA group), 5 cases with progressive supranuclear palsy (all in the nfvPPA group), 5 cases with Pick’s disease (4 in the nfvPPA group and 1 in the svPPA group), 2 cases with FTLD-TDP type A (all in the GRN-PPA group), 1 case with FTLD-TDP type B (in the svPPA group), 1 case with FTLD-TDP type C (in the svPPA group) and 1 case with an unclassifiable tauopathy (in the nfvPPA group)

†Different between all PPA group and HC group (*P*<.05)

‡Different between lvPPA, nfvPPA, and PPA-GRN (*P*<.05, Bonferroni adjusted)

§Different between svPPA and nfvPPA (*P*<.05, Bonferroni adjusted)

As shown in Table 1, 29 (14%) of the participants with PPA underwent autopsy evaluation. All the participants with a neuropathological diagnosis of Alzheimer’s disease (*n* = 8) had a diagnosis of lvPPA. Conversely, all participants with a diagnosis of FTLD were classified in the nfvPPA (*n* = 16), svPPA (*n* = 3), and GRN-PPA (*n* = 2) groups.

**Fig. 2:**
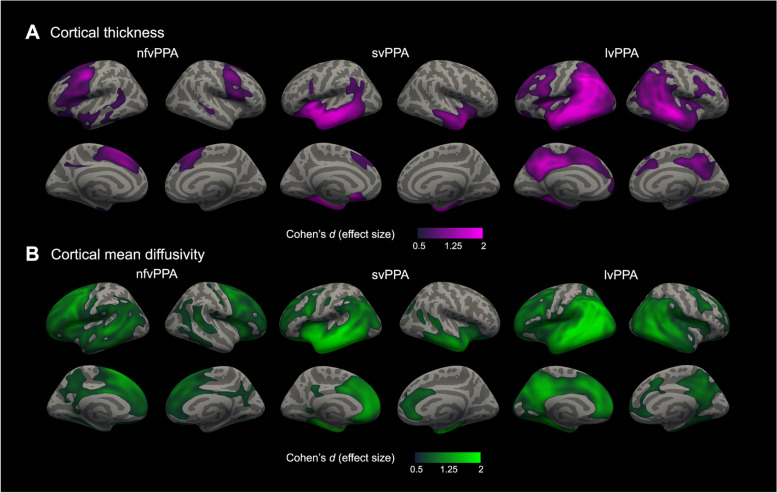
Group comparison of cortical thickness and cortical mean diffusivity between participants with PPA and healthy controls. Statistically significant results between major PPA subtypes (52 nfvPPA, 31 svPPA, 32 lvPPA) and healthy controls (*n* = 89) for cortical thickness (**A**) and cortical mean diffusivity (**B**). Cortical thickness analyses were adjusted for age, sex, handedness, and MRI scan. Cortical mean diffusivity analyses were adjusted for age, sex, and handedness after a harmonization step. Only effect sizes (Cohen’s *d*) inside clusters that survived family-wise error correction (*P*<.001) are shown. Abbreviations: lvPPA, logopenic variant of primary progressive aphasia; nfvPPA, non-fluent/agrammatic variant of primary progressive aphasia; svPPA, semantic variant of primary progressive aphasia

We first compared cortical thickness and cortical mean diffusivity between PPA and controls. As shown in Fig. 2, the nfvPPA group showed the expected pattern of cortical thinning in the precentral, inferior, middle, and superior frontal gyri as well as supplementary motor and dorsomedial prefrontal cortex in both hemispheres, but higher effect sizes were observed in the left hemisphere. In this group, the cortical mean diffusivity map involved more regions, including the frontal pole, the insula, dorsal anterior and pregenual cingulate cortex, posterior cingulate and precuneus, and the temporo-parietal junction of both hemispheres as well as a large swatch of the left lateral temporal cortex (Fig. 2B). The svPPA showed the expected pattern of cortical thinning involving the lateral, ventral, and medial anterior temporal cortex and temporal pole, insula, subgenual anterior cingulate cortex, inferior parietal lobule, and caudal inferior frontal gyrus of the left hemisphere and, to a lesser extent, the anterior temporal cortex and insula of the right hemisphere (Fig. 2A). Cortical mean diffusivity changes were present in the same regions as thickness changes but extended beyond those to include the pregenual and dorsal anterior cingulate cortex of both hemispheres and the dorsolateral prefrontal cortex and the inferior parietal lobule of the left hemisphere (Fig. 2B). Participants within the lvPPA group displayed cortical thinning in the expected lateral temporal and inferior parietal lobules of both hemispheres, with additional thinning in superior parietal lobules, prefrontal cortex, and posterior cingulate and precuneus cortex, with higher effect sizes in the left hemisphere (Fig. 2A). Again, in the lvPPA variant, we also observed more widespread cortical mean diffusivity increases involving those same regions as well as the medial prefrontal cortex and pregenual and dorsal anterior cingulate cortex of both hemispheres (Fig. 2B).

**Fig. 3:**
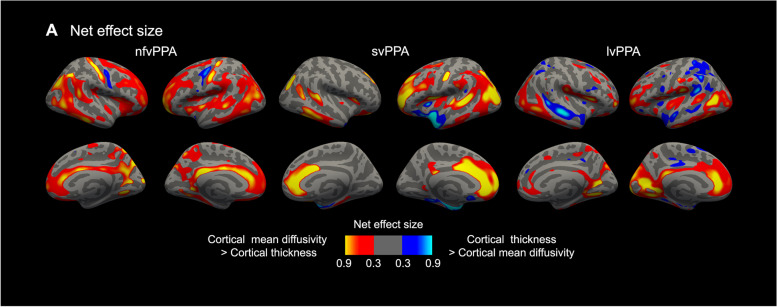
Net effect size. The net effect size (**A**) was obtained by subtracting the cortical thickness and cortical mean diffusivity effect size maps of the comparison between PPA participants and healthy controls. The red-yellow color represents cortical areas where the cortical mean diffusivity has a higher effect size than cortical thickness. The blue color represents cortical areas where the cortical thickness has a higher effect size than cortical mean diffusivity. Abbreviations: lvPPA, logopenic variant of primary progressive aphasia; nfvPPA, non-fluent/agrammatic variant of primary progressive aphasia; svPPA, semantic variant of primary progressive aphasia

Thus, while cortical thickness and cortical mean diffusivity maps showed a partial overlap in all PPA variants, cortical mean diffusivity changes with at least moderate effect sizes extended beyond the areas of cortical thinning (Fig. 3). Importantly, the effect sizes of cortical mean diffusivity in overlapping areas were higher than the effect sizes of cortical thickness with the sole exception of neurodegeneration cores for each PPA variant (namely, the left temporal pole for svPPA, premotor cortex for nfvPPA, and temporo-parietal cortex for lvPPA). Moreover, we observed essentially the same patterns of cortical thickness and cortical mean diffusivity changes when each cohort was analyzed separately (data not shown).

**Fig. 4:**
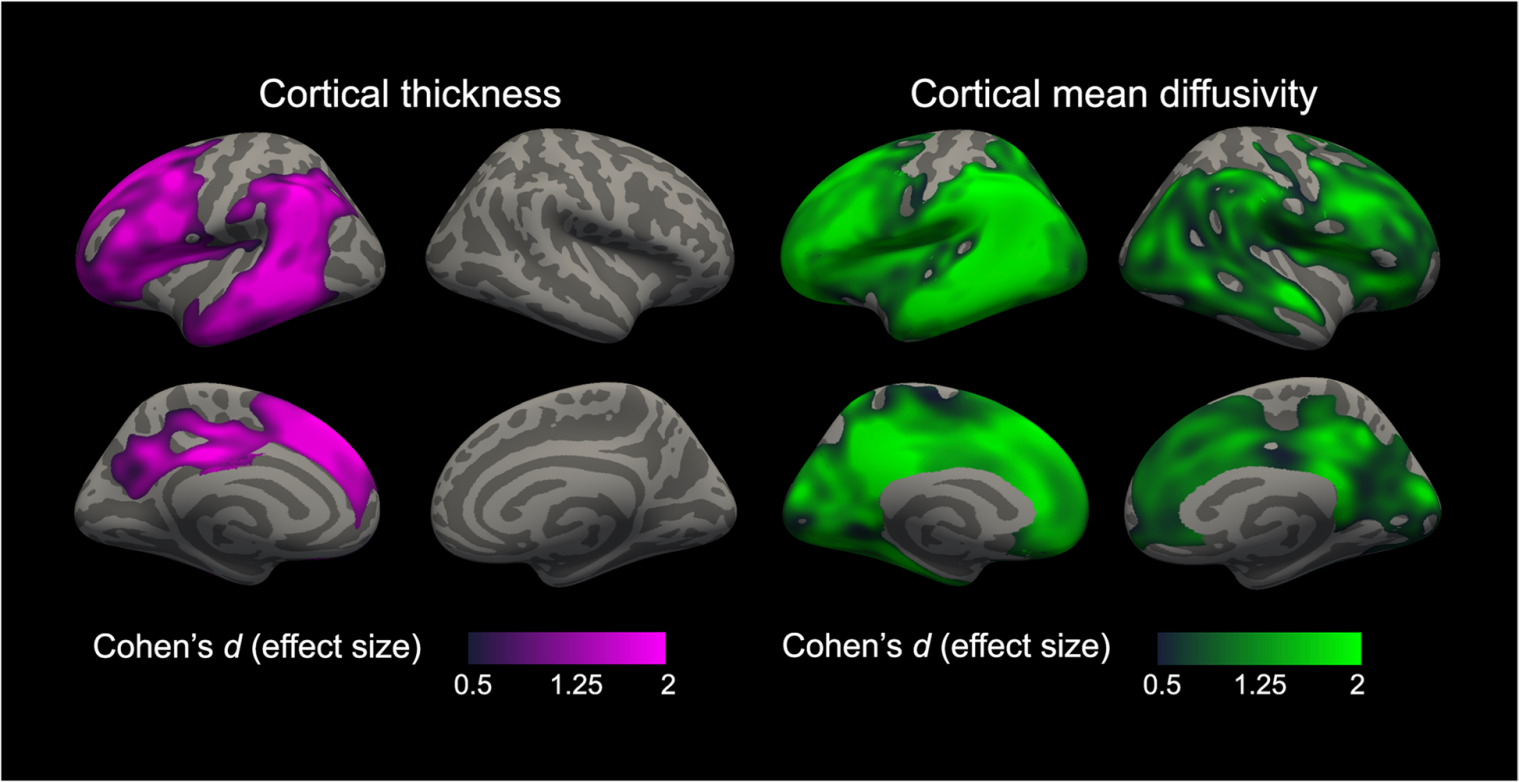
Group comparison of cortical thickness and cortical mean diffusivity between participants with *GRN* mutation and healthy controls. Statistically significant results between PPA participants with *GRN* mutation (*n* = 5) and healthy controls for cortical thickness (**left**) and cortical mean diffusivity (**right**). For these analyses, we only considered healthy controls scanned at the same MRI than PPA participants with *GRN* mutation (*n* = 38). Cortical thickness analyses were adjusted for age, sex, handedness, and MRI scan. Cortical mean diffusivity analyses were adjusted for age, sex, and handedness after a harmonization step. Only effect sizes (Cohen’s *d*) inside clusters that survived family-wise error correction (*P<*.05) are shown

Figure 4 shows cortical thickness and cortical mean diffusivity comparison between PPA participants with *GRN* mutation and controls. As expected, we observed cortical thinning in the prefrontal, lateral temporal, and inferior parietal cortex. In this group, cortical thinning was restricted to the left hemisphere. In the PPA-*GRN* group, we also observed widespread increases in cortical mean diffusivity affecting both hemispheres (Fig. 4).

**Fig. 5:**
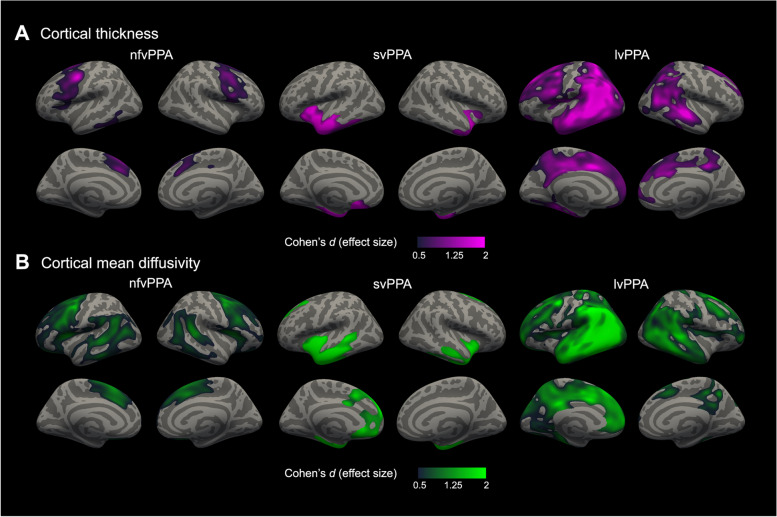
Group comparison of cortical thickness and cortical mean diffusivity between participants with mild PPA and healthy controls. Statistically significant results between participants with mild PPA (as defined by a global CDR® plus NACC FTLD score of 0.5; 20 nfvPPA, 6 svPPA, and 9 lvPPA) and healthy controls for cortical thickness (**A**) and cortical mean diffusivity (**B**). Cortical thickness analyses were adjusted for age, sex, handedness, and MRI scan. Cortical mean diffusivity analyses were adjusted for age, sex, and handedness after a harmonization step. Only effect sizes (Cohen’s *d*) inside clusters that survived family-wise error correction (*P*<.001) are shown. Abbreviations: lvPPA, logopenic variant of primary progressive aphasia; nfvPPA, non-fluent/agrammatic variant of primary progressive aphasia; svPPA, semantic variant of primary progressive aphasia

Next, we explored group differences in cortical thickness and cortical mean diffusivity in the subgroup of participants with early-stage PPA (global CDR® plus NACC FTLD score of 0.5). As expected, we observed the same pattern of cortical thinning and cortical mean diffusivity increases. However, these changes were more restricted to the neurodegeneration cores of each PPA variant (Fig. 5).

**Fig. 6:**
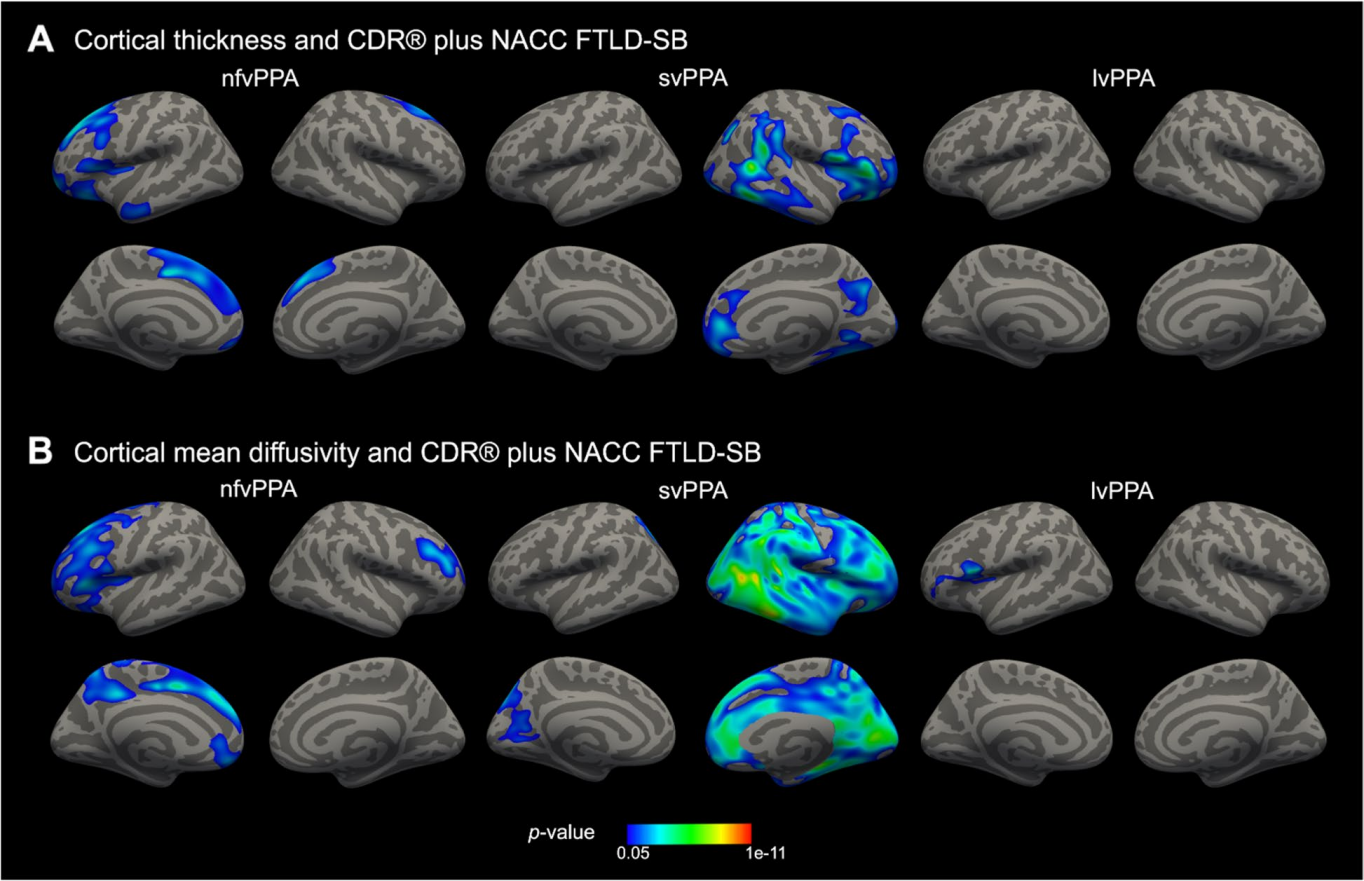
Correlation of cortical thickness and cortical mean diffusivity with the CDR® plus NACC FTLD-SB. Relationship of cortical thickness (**A**) and cortical mean diffusivity (**B**) with the CDR® plus NACC FTLD scores. The CDR® plus NACC FTLD-SB scores were negatively correlated with cortical thickness and positively correlated with cortical mean diffusivity. Cortical thickness analyses were adjusted for age, sex, handedness, and MRI scan. Mean diffusivity analyses were adjusted for age, sex, and handedness after a harmonization step. Only clusters that survived family-wise error correction (*P* < .05) are shown. Abbreviations: CDR, clinical dementia rating; CDR® plus NACC FTLD-SB, CDR Dementia Staging Instrument plus National Alzheimer’s Coordinating Center Behavior and Language Domains sum of boxes; lvPPA, logopenic variant of primary progressive aphasia; nfvPPA, non-fluent/agrammatic variant of primary progressive aphasia; svPPA, semantic variant of primary progressive aphasia

Finally, we evaluated the capacity of cortical thickness and cortical mean diffusivity to reflect disease severity in PPA as measured by the CDR® plus NACC FTLD-SB scores. As shown in Fig. 6, we observed an inverse correlation between CDR® plus NACC FTLD-SB scores and cortical thickness in left-lateralized prefrontal regions in nfvPPA, widespread regions of the right frontal and temporal cortex in svPPA, and no localizable effects in lvPPA. As with our other effects, we observed larger clusters with similar localization between cortical mean diffusivity and CDR® plus NACC FTLD-SB scores (Fig. 6). We observed similar results when studying the correlation of cortical thickness and cortical mean diffusivity with MMSE (Supplementary Fig. 1).

## Discussion

This large multicenter study investigated cortical mean diffusivity in PPA. Importantly, we showed that cortical mean diffusivity increases not only coincided with areas that showed cortical thinning but also involved other areas that typically become affected later during disease progression. Cortical mean diffusivity was increased in patients with very mild PPA when only minimal cortical thinning was observed. Finally, we also explored the correlation between cortical mean diffusivity and clinical measures of disease severity and general cognitive function. Taken together, these findings suggest that cortical mean diffusivity might be more sensitive than cortical thickness to detect the earliest disease-related cortical changes in PPA.

Cortical mean diffusivity has been recently proposed as a sensitive biomarker for the detection of the earliest cortical changes in sporadic Alzheimer’s disease and the Amyotrophic Lateral Sclerosis-Frontotemporal dementia continuum [13, 14, 16–18]. This study expands these findings to PPA. We showed that cortical mean diffusivity increases spread beyond the areas of cortical thinning in PPA. Most previous studies using diffusion tensor imaging in PPA patients have focused on the white matter, probably because of the technical difficulties in the study of cortical microstructure. One study found overlapping patterns between atrophy and increases in cortical mean diffusivity in the nfvPPA and semantic dementia [39]. Our study builds on these results using a larger sample, a surface-based approach, and the inclusion of all PPA variants included in the 2011 consensus criteria. Consequently, we were able to explore the added value of cortical mean diffusivity over cortical thickness, particularly in mildly symptomatic cases.

PPA variants are characterized by a focal onset of neurodegeneration spreading along specific networks [40]. We observed cortical thinning including the core of neurodegeneration of each PPA when comparing cortical thickness and cortical mean diffusivity of PPA participants and controls. Importantly, we also found cortical mean diffusivity increases beyond the regions with atrophy in regions that are known to become involved with disease progression. The effect size maps showed moderate to high net effect size favoring cortical mean diffusivity in key regions for each PPA subtype except in the neurodegenerative core of each syndrome. Although the origin of these microstructural changes is unclear, higher cortical mean diffusivity values have been found to reflect a loss of tissue integrity and breakdown of cell membranes in the cortex. We also hypothesize that cortical mean diffusivity might reach floor effects earlier than atrophy. Hence, the very focal atrophy seen at the epicenters in PPA (particularly in the svPPA variant) [11] is better reflected with cortical thickness, but the spread of pathology to other cortical areas may be better captured by cortical mean diffusivity.

Furthermore, we performed an exploratory analysis in the PPA participants with *GRN* mutations. We classified PPA participants with *GRN* mutations in a separate group because these cases are characterized by a more widespread and asymmetric pattern of gray matter loss. In the PPA-*GRN* group, cortical thinning was restricted to the left hemisphere, but cortical mean diffusivity extended to both hemispheres. This finding also supports the view that cortical mean diffusivity is capturing early neurodegenerative changes antedating overt neuronal loss and cortical thinning. This observation in the PPA-*GRN* group also encourages the investigation of cortical mean diffusivity in larger samples of FTLD mutation carriers.

The suggestion that cortical mean diffusivity may be more sensitive than cortical thickness to detect the PPA cortical changes is further supported by our correlation analyses with the CDR® plus NACC FTLD-SB and MMSE scores. The CDR® plus NACC FTLD-SB has been validated as a tool for disease monitoring in clinical trials [28, 41]. Although the CDR® plus NACC FTLD-SB scores correlated with cortical thickness in some small frontotemporal clusters, we found a stronger and more widespread correlation with cortical mean diffusivity. These results should be, however, considered exploratory. More studies are needed to determine the precise relationship between cortical mean diffusivity and FTLD-related neurodegeneration, from the preclinical to the dementia stage.

Although clinical-pathological correlations are not perfect, each PPA variant is associated with typical pathologies. For instance, in the absence of a *GRN* mutation, the nfvPPA syndrome is frequently associated with Tau subtypes of FTLD, while the svPPA is associated with the TDP-C subtype of FTLD, and lvPPA is typically associated with AD pathologic changes [42]. We observed similar cortical mean diffusivity increases for each PPA group, suggesting that cortical mean diffusivity changes may be an unspecific biomarker of neurodegeneration. However, the difference between cortical thickness and cortical mean diffusivity changes was less clear in the lvPPA group. In addition, the correlation between cortical mean diffusivity and disease severity was also less evident in the lvPPA group than in the nfvPPA and svPPA groups. Two important factors should be considered when interpreting these findings. First, the pattern of neurodegeneration in the lvPPA variant is more widespread and more heterogeneous than the pattern observed in other PPA variants, as noted by previous studies [43]. Importantly, a higher heterogeneity within a neurodegenerative syndrome penalizes finding statistically significant correlations between general measures of disease severity and neuroimage measures [15]. Second, the lvPPA syndrome is strongly associated with Alzheimer’s disease, a disease characterized by the extraneuronal deposition of fibrillary amyloid, together with intraneuronal aggregation of tau. Of note, we have previously shown that amyloid deposition impacts cortical mean diffusivity, even in the preclinical phase of Alzheimer’s disease [44]. Because only tau pathology (and not amyloid deposition) is closely correlated with neurodegeneration in Alzheimer’s disease, we hypothesize that amyloid-related microstructural changes could attenuate the association between disease severity and cortical mean diffusivity in the lvPPA group [45]. However, additional multimodal imaging studies are needed to confirm this hypothesis and precise the relationship between amyloid deposition, tau pathology, and cortical microstructure in Alzheimer’s disease. In addition, autopsy-proven studies should explore the added value of cortical mean diffusivity (alone or combined with white matter diffusion tensor imaging changes) to detect specific neuropathological signatures (i.e., prominent microstructural changes in the subcortical white matter in FTLD-Tau or widespread gray matter changes in Alzheimer’s disease with relative sparing of subcortical white matter) [46–48].

Our findings support the role of cortical mean diffusivity as a potential neurodegeneration biomarker in PPA to be used in clinical trials. Trials of drugs targeting abnormal protein deposition need meaningful end points. Historically, these end points included neuropsychological scores and functional scales. Brain imaging could provide more sensitive and robust evidence of disease modification [49]. Our study shows that cortical mean diffusivity is a more sensitive biomarker than cortical thickness and correlates more strongly with clinical and cognitive measures, in agreement with a previous report [49]. The increased sensitivity of cortical mean diffusivity suggests it may be a sensitive tool suitable to monitor the earliest cortical changes in preclinical or mildly symptomatic phases of FTLD [50].

The main strengths of this study are the relatively large number of participants with PPA at a mild disease stage, and the surface-based analyses using a previously validated technique. This surface-based approach solves some of the limitations and methodological concerns that have been previously reported when using a voxel-based approach [37]. Moreover, we enriched our description of the cortical mean diffusivity in PPA and we were able to replicate our results in two different cohorts.

### Limitations

This study has also some limitations. A proportion of participants with PPA were excluded due to segmentation or diffusion tensor imaging processing errors. Even though this is an inherent limitation of our surface-based approach, future improvements in T1 MRI acquisitions or the use of higher field MRIs will likely reduce the number of subjects excluded due to segmentation errors. Also, both the PPA-*GRN* group and the subgroup of PPA participants at a mild disease stage were small. Finally, further longitudinal studies in presymptomatic mutation carriers should confirm that cortical mean diffusivity changes track disease progression and antedate cortical atrophy in patients with PPA.

## Conclusions

Cortical mean diffusivity shows promise as a sensitive biomarker for the study of the earliest neurodegeneration-related cortical changes in PPA. Further longitudinal studies including preclinical mutation carriers are needed to fully determine the potential utility of this biomarker to detect cortical changes antedating cerebral atrophy at the earliest stages of the disease and to track disease progression.

## Supplementary Information

**Additional file 1.** Supplementary material.

## Data Availability

The datasets analyzed during the current study are available from the corresponding authors on reasonable request.

## Abbreviations

AD: Alzheimer’s disease
CATFI: Catalan Frontotemporal Dementia Initiative
CDR: Clinical Dementia Rating
CDR® plus NACC FTLD-SB: CDR Dementia Staging Instrument plus National Alzheimer’s Coordinating Center Behavior and Language Domains sum of boxes
FDR: False discovery rate
FWE: Family-wise error
HC: Healthy control
MMSE: Mini-Mental State Examination
MRI: Magnetic resonance image
PPA: Primary progressive aphasia
UCSF: University of California San Francisco
