# Correction: Cortical microstructure in primary progressive aphasia: a multicenter study

**DOI:** 10.1186/s13195-023-01179-9

**Published:** 2023-02-04

**Authors:** Ignacio Illán-Gala, Victor Montal, Sergi Borrego-Écija, Maria Luisa Mandelli, Neus Falgàs, Ariane E. Welch, Jordi Pegueroles, Miguel Santos-Santos, Alexandre Bejanin, Daniel Alcolea, Oriol Dols-Icardo, Olivia Belbin, Mª. Belén Sánchez-Saudinós, Nuria Bargalló, Sofía González-Ortiz, Albert Lladó, Rafael Blesa, Bradford C. Dickerson, Howard J. Rosen, Bruce L. Miller, Alberto Lleó, Maria Luisa Gorno-Tempini, Raquel Sánchez-Valle, Juan Fortea

**Affiliations:** 1 Memory Unit, Department of Neurology, Hospital de la Santa Creu i Sant Pau, Biomedical Research Institute Sant Pau, Sant Antoni Maria Claret, 167, 08025 Barcelona, Spain; 2 Centro de Investigación Biomédica en Red de Enfermedades Neurodegenerativas (CIBERNED), Barcelona, Spain; 3 Atlantic Fellow for Equity in Brain Health at the University of California San Francisco, San Francisco, CA 94115 USA; 4 Alzheimer’s Disease and Other Cognitive Disorders Unit, Service of Neurology, Hospital Clínic de Barcelona, Institut d’Investigació Biomèdica August Pi i Sunyer, University of Barcelona, 08036 Barcelona, Spain; 5 Memory and Aging Center, Department of Neurology, University of California, San Francisco, CA 94115 USA; 6 Radiology Department, Hospital Clinic Barcelona and Magnetic Resonance Image Core facility, Institut d’Investigacions Biomèdiques August Pi i Sunyer (IDIBAPS), Barcelona, Spain; 7 Department of Radiology, Hospital del Mar, Barcelona, Spain; 8 Department of Neurology, Massachusetts General Hospital and Harvard Medical School, Boston, MA USA; 9 Massachusetts Alzheimer’s Disease Research Center, Boston, MA USA; 10 Barcelona Down Medical Center. Fundació Catalana de Síndrome de Down, Barcelona, Spain

**Correction: Alz Res Therapy 14, 27 (2022)**

10.1186/s13195-022-00974-0

Following publication of the original article [1], the authors realized that a critical funding agency was not included in the funding section. The funding information that should be included: "European Union's Horizon 2020, 'MES-CoBraD' (H2020-SC1-BHC-2018-2020/GA 965422 to JF)”.

The original article [1] has been updated.
