# Supplementary material for: Cortical microstructure in primary progressive aphasia: a multicenter study
Source: Alzheimers Res Ther. 2022 Feb 9;14:27. doi: 10.1186/s13195-022-00974-0 (PMC8830043; doi:10.1186/s13195-022-00974-0)
Supplement: Supplementary file 1 — Additional file 1. Supplementary material. [file 13195_2022_974_MOESM1_ESM.docx]

**Supplementary material**

**Supplementary Methods**

**Monte Carlo simulation**

In this work we used a Monte Carlo simulation with 10000 repeats as implemented in Freesurfer to correct for false positives (Hagler *et al.*, 2006) . Briefly, this cluster-extension-based method is based in computing the probability that a certain cluster of a specific size is obtained by chance/noise. This probability is computed using Monte Carlo simulations where: 1) white Gaussian noise is synthesized in the Freesurfer’s *fsaverage* standard space surface, 2) the noise is smoothed by a certain FWHM (data-dependent), 3) the smoothed values are thresholded using a priori threshold (here 1.3 and 3 related to 0.05 and 0.001) and 4) the maximum cluster size for the simulation is recorded. These steps are repeated 10000 times in order to generate a distribution of maximum cluster size generated by random noise. If our statistically significant cluster size is NOT smaller than the obtained during the Monte Carlo simulation more than 500 iterations (p < 0.05), the software considers that the cluster is not given by chance and survived multiple comparisons. This distribution is provided by Freesurfer (specifically, in the command mri_surfcluster)”.

**Supplementary references**

Hagler DJ, Saygin AP, Sereno MI. Smoothing and cluster thresholding for cortical surface-based group analysis of fMRI data. NeuroImage 2006; 33: 1093–103.

**Supplementary Table 1. Structural T1-weighted image acquisition protocols by center.**

|  | CATFI I | CATFI II | CATFI III | UCSF I | UCSF II |
| --- | --- | --- | --- | --- | --- |
| Manufacturer  (system) | Philips (Achieva) | Philips (Achieva) | Siemens (Tim Trio) | Siemens  (Tim Trio) | Siemens  (Magnetom Prisma) |
| Magnet strength | 3T | 3T | 3T | 3T | 3T |
| Repetition time (ms) | 8.1 | 6.74 | 2300 | 2300 | 2300 |
| Echo time (ms) | 3.7 | 3.14 | 2.98 | 2.98 | 2.98 |
| Slice Thickness (mm) | 1 | 1. 2 | 1 | 1 | 1 |
| Voxel size (mm) | 0.94 x 0.94 x 1 | 0.9 x 0.9 x 1.2 | 1 x 1 x 1 | 1 x 1 x 1 | 1 x 1 x 1 |
| Number of participants scanned,  n (%) | 13 (6%) | 37 (18%) | 51 (24%) | 62 (30%) | 46 (22%) |

**Supplementary Table 1. Structural T1-weighted image acquisition protocols by center.**

**Abbreviations:** CATFI=Catalan frontotemporal dementia initiative; mm = millimeters; ms = milliseconds**;** UCSF=university of California san Francisco

**Supplementary Table 2. Diffusion weighted image acquisition protocols by center.**

|  | CATFI I | CATFI II | CATFI III | UCSF I | UCSF II |
| --- | --- | --- | --- | --- | --- |
| Manufacturer  (system) | Philips (Achieva) | Philips (Achieva) | Siemens (Tim Trio) | Siemens  (Tim Trio) | Siemens  (Magnetom Prisma) |
| b-value (s/mm^2^) | 1000 | 800 | 1000 | 1000 | 2000 |
| Number of directions | 32 | 15 | 30 | 44 | 64 |
| Phase encoding direction | Anterior-Posterior | Anterior-Posterior | Anterior-Posterior | Anterior-Posterior | Anterior-Posterior |
| Repetition Time (ms) | 13677 | 6672 | 7700 | 9200 | 2300 |
| Echo Time (ms) | 61 | 60 | 89 | 31 | 32.4 |
| Slice Thickness (mm) | 2 | 2 | 2 | 2.7 | 2.2 |
| Voxel Size (mm) | 2 x 2 x 2 | 1.64 x 1.64 x 1.64 | 2 x 2 x 2 | 2.7 x 2.7 x 2.7 | 2.2 x 2.2 x 2.2 |
| Number of participants scanned, n (%) | 13 (6%) | 37 (18%) | 51 (24%) | 62 (30%) | 46 (22%) |

**Supplementary Table 1. Structural T1-weighted image acquisition protocols by center.**

**Abbreviations:** CATFI=Catalan frontotemporal dementia initiative; mm = millimeters; ms = milliseconds**;** UCSF=university of California san Francisco

**Supplementary Figure 1. Correlation of cortical thickness and cortical mean diffusivity with Mini-Mental State Examination**

**
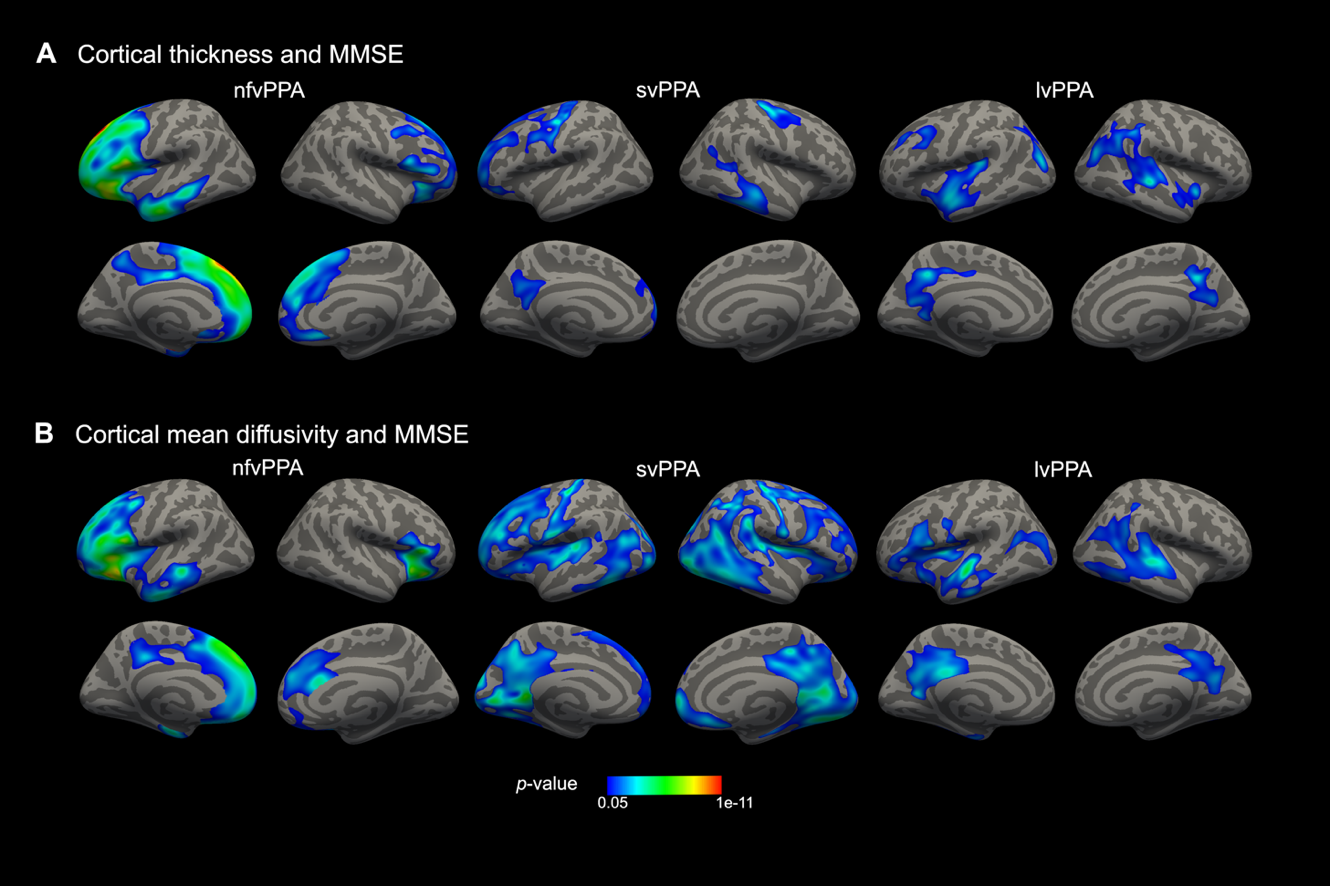
**

**Supplementary Figure 1.** Relationship of cortical thickness (A) and cortical mean diffusivity (B) with the MMSE scores. The MMSE scores was positively correlated with cortical thickness and negatively correlated with cortical mean diffusivity. Cortical thickness analyses were adjusted for age, sex, handedness, and MRI scan. Mean diffusivity analyses were adjusted for age, sex, and handedness after a harmonization step. Only clusters that survived familywise error correction (*P* < .05) are shown.

**Abbreviations:** lvPPA=logopenic variant of primary progressive aphasia; MMSE=mini-mental state examination; nfvPPA=non-fluent/agrammatic variant of primary progressive aphasia; svPPA=semantic variant of primary progressive aphasia.
